# “Your Body Feels Better When You Drink Water”: Parent and School-Age Children’s Sugar-Sweetened Beverage Cognitions

**DOI:** 10.3390/nu10091232

**Published:** 2018-09-05

**Authors:** Kaitlyn M. Eck, Aleksandr Dinesen, Elder Garcia, Colleen L. Delaney, Oluremi A. Famodu, Melissa D. Olfert, Carol Byrd-Bredbenner, Karla P. Shelnutt

**Affiliations:** 1 Department of Nutritional Sciences, Rutgers University, 26 Nichol Avenue, New Brunswick, NJ 08901, USA; ard180@sebs.rutgers.edu (A.D.); colleenldelaney90@gmail.com (C.L.D.); bredbenner@sebs.rutgers.edu (C.B.-B.); 2 Department of Family, Youth, and Community Sciences, University of Florida, Gainesville, FL 32611, USA; elder89@ufl.edu (E.G.); oluremifamodu@tcomn.com (O.A.F.); kpagan@ufl.edu (K.P.S.); 3 Division of Animal and Nutritional Sciences, West Virginia University, 1194 Evansdale Dr. G28, West Virginia University, Morgantown, WV 26506, USA; Melissa.Olfert@mail.wvu.edu

**Keywords:** sugar-sweetened beverages, children, parents, social cognitive theory, nutrition education, health promotion

## Abstract

Sugar-sweetened beverages (SSBs) are a leading source of added sugar in the American diet. Further, ingestion of added sugars from SSBs exceeds recommendations. Thus, interventions that effectively reduce SSB consumption are needed. Focus group discussions with parents (*n* = 37) and school-aged children between the ages of 6 and 11 years (*n* = 41) from Florida, New Jersey, and West Virginia were led by trained moderators using Social Cognitive Theory as a guide. Trends and themes that emerged from the content analysis of the focus group data indicated that both parents and children felt that limiting SSBs was important to health and weight control. However, parents and children reported consuming an average of 1.85 ± 2.38 SD and 2.13 ± 2.52 SD SSB servings/week, respectively. Parents and children were aware that parent behaviors influenced kids, but parents reported modeling healthy SSB behaviors was difficult. Busy schedules, including more frequent parties and events as children get older, were another barrier to limiting SSBs. Parents were most successful at limiting SSBs when they were not in the house. This qualitative research provides novel insights into parents’ and children’s cognitions (e.g., beliefs, attitudes), barriers, and facilitators related to SSB ingestion. Consideration of these insights during nutrition intervention development has the potential to improve intervention effectiveness in reducing SSB intake.

## 1. Introduction

Sugar sweetened beverages (SSBs) are a leading source of added sugars in the American diet [1]. Ingestion of these beverages is positively associated with excess body weight [2,3,4] as well as an increased risk of metabolic syndrome and its associated conditions, including cardiovascular disease and type 2 diabetes [5,6,7,8]. Currently, 20% of school-aged children (6 to 11 years) in the United States are classified as obese [9]. The Academy of Nutrition and Dietetics has identified SSBs as a contributor to childhood overweight and obesity; hence, this organization’s Pediatric Weight Management Guidelines recommend limited intake of these beverages by children [10].

The Dietary Guidelines for Americans recommend keeping added sugar intake to less than 10% of an individual’s daily calorie intake [11]; however, many Americans exceed this advice [1,12,13,14]. For instance, nearly 66% of school-aged children are consuming at least one SSB each day and as a result they are consuming over 7% of their total daily calories from these beverages [14]. Sugary drinks provide 60% of the total calories from added sugar in the diets of children in the U.S. [15]. 

In addition to its links to obesity and related physical and mental health effects [16,17,18], sugary beverages have negative consequences on oral health in children [19,20]. SSBs also displace nutrient-dense beverages, especially dairy milk [21,22], a key source of calcium, potassium, and vitamin D in the diets of American children [23]. Additionally, there is a positive relationship between SSB intake and weakened bones and increased risk of fractures [24].

Several studies have attempted to elucidate factors influencing SSB consumption. One factor is family behaviors, with a positive correlation between parent and child SSB intake [25,26,27], which is indicative of the observational learning construct of the Social Cognitive Theory [28,29]. Another factor affecting SSB intake is behavioral facilitators [28,29,30], also a Social Cognitive Theory construct. Environmental conditions [28,29,30] where there is greater SSB availability in the home, appear to facilitate SSB drinking [25,26,31]. Another environmental condition promoting consumption of sugary drinks is location of eating—as the frequency of eating away from home rises, so does SSB intake [32].

In recent years, consumption of sugary drinks has declined [33]. However, there is considerable opportunity to further reduce intake of these calorie-dense, nutrient-bare beverages. A greater understanding of factors supporting SSB consumption as well as those that help reduce it could lead to more effective behavior change and obesity prevention programs. Thus, this study aimed to qualitatively explore factors affecting SSB intake with the goal of creating recommendations that inform the development of health promotion and obesity prevention materials predicated on the Social Cognitive Theory that aim to reduce sugary drink intake of parents and school-aged children (6 to 11 years).

## 2. Materials and Methods

The Institutional Review Boards at the authors’ universities approved the procedures for this investigation. All parents gave informed consent for their children as well as themselves. Prior to data collection, children gave verbal assent.

### 2.1. Sample

Parents who resided in Florida, New Jersey, or West Virginia and had one or more children aged 6 to 11 years were recruited to enroll in focus groups discussing home and lifestyles changes to promote child health and development. Electronic (website, emails) and paper recruitment announcements were distributed at workplaces, schools, community centers, and other locations frequented by parents. Recruitment announcements were in English and Spanish and specified that focus groups would take about 60 min and that participants would receive $25. The announcements also invited parents to sign up their school-age children for a 30-min focus group discussion and indicated child participants would receive $15. Parents and children participating in the focus groups were not necessarily related. Focus group size was intentionally kept small to permit everyone to fully participate.

### 2.2. Instruments

Before focus groups began, parents completed a brief survey to report demographic characteristics (e.g., age, highest education level) and frequency of SSB intake. Children completed a similar form.

A semi-structured questionnaire based on key Social Cognitive Theory (SCT) constructs (i.e., attitudes toward SSB, barriers to reducing SSB intake, and facilitators for reducing SSB intake) was developed. SCT considers both inter- and intra-personal factors influencing behavior performance including self-efficacy, collective efficacy (belief that a group is able to perform a behavior change), and observational learning (learning by watching others perform a behavior) [28,29,30], which makes it suitable to employ in family-based interventions. Furthermore, SCT addresses the interaction between people and their environment—a bidirectional influence between these that is referred to as reciprocal determinism [28,29,30]. The notion of reciprocal determinism also makes SCT a good fit for home-based interventions where family members’ behaviors affect and are affected by their home environment.

Prior to commencing the study, all data collectors were trained to conduct the focus groups in a uniform manner using standard procedures [34]. English and Spanish focus groups were conducted separately to accommodate the parents’ language preference. All child focus groups were in English because all were fluent in this language. Younger children (ages 6 to 9) participated in focus groups separate from those comprised of older, more mature children (ages 9 to 11). At each focus group, a second trained researcher took comprehensive notes of the discussion and transcribed the notes within 48 h of the focus group, including translating Spanish language focus group notes. The researcher moderating the focus group reviewed the notes and discussed them with the note-taker to ensure they were a clear, complete, and correct record.

### 2.3. Data Analysis

Descriptive statistics summarizing data collected in the survey administered before the focus group were conducted were calculated using SPSS version 21.0 (Chicago, IL, USA). Three researchers trained in standard content analysis procedures independently categorized focus group data into themes (Miles and Huberman 1994 [35], Harris, Gleason et al. 2009 [36]). Content analysis generates objective, systematic descriptions [37] that permit drawing “replicable and valid inferences from the data to their context” [38]. The independent content analysis findings were compared and researchers discussed them to reach consensus. Focus group data were continually analyzed during the data collection period to determine when no new information was being revealed (i.e., data saturation was reached) and data collection could end [39].

## 3. Results

A total of 37 parents (97% female) participated in 1 of 13 focus group discussions about beverages. Three of these focus groups were performed in Florida (*n* = 13 parents), 5 in New Jersey (*n* = 12 parents), and 4 in West Virginia (*n* = 12 parents). Parents were 36.3 ± 5.13 SD years old and had 2.42 ± 0.91 SD children under 18 living at home. The group was well educated, with 57% having obtained a baccalaureate degree or higher. Two-thirds of the parents completed the focus groups in English, with the remainder in Spanish.

A total of 41 school-aged children (ages 6 to 11 years) participated in 1 of 15 focus group discussions about beverages. Five of these focus groups were performed in Florida (*n* = 12 children), 5 in New Jersey (*n* = 13 children), and 5 in West Virginia (*n* = 16 children). The average age of the children was 8.46 ± 1.85 SD years. Participants had 1.17 ± 2.645 SD older siblings and 1.50 ± 1.57 SD younger siblings.

### 3.1. Parent Focus Groups

Survey results illustrated that parents consumed an average of 1.85 ± 2.38 SD SSBs per week. A comparison of SSB intake by language spoken and state of residence indicated intake was similar. Additionally, no differences were discerned in focus group data by language or state of residence, so data are presented in aggregate.

#### 3.1.1. Parents’ Attitudes toward SSB Consumption

The majority of parents felt that limiting sugary beverages like soft drinks is “very important” for their children’s overall health and wellbeing commenting, “young kids can have health problems from the sugar”. Parents recognized the value of reducing SSB consumption, indicating that “sugary drinks have empty calories, no vitamins, and lack the ability to keep you full” and these drinks “can affect kids so much that children become overweight”. One noted, “I feel like, if I knew what I know now at their age, I wouldn’t have had to get a gastric sleeve”. On the other hand, another commented “I don’t try to tell them (kids) about gaining weight because I don’t want them to think about body images at their age”.

Another common concern was oral health, with parents commenting on the “effect [SSBs have on] their teeth”, with others extending the effects to complexion and other physical aspects—“it’s really a whole body thing”. Other health conditions, such as kidney stones and diabetes, were mentioned by parents who had a family history of these conditions.

Behavior and adequate sleep were common themes in focus groups, with parents observing that “I see a palpable change in my kids when they are sugared up” and “it disrupts sleeping habits”. Numerous respondents indicated that SSBs made their children “hyperactive”, “bounce off the walls”, and “rowdier”.

Some participants alluded to “addictive” qualities of these drinks. “The more sugar they start to take in, the more sugar they want”. Caffeine also was of concern to some parents who indicated that they “try to limit their caffeine intake so they (kids) don’t get addicted to it” and “kids don’t need the caffeine”.

A few commented on environmental/geographical influences on beverage intake. One parent said, “in southern West Virginia, soda is normal, and it was difficult to change when I realized that it wasn’t normal and healthy”.

#### 3.1.2. Parents’ Perceived Barriers to Limiting SSB Consumption

A common barrier to limiting children’s SSB intake was parental role modeling. Most parents reported that their children commonly want to partake in whatever their mother or father do: “they see an example and they imitate it" and “if I consume it, they are going to consume it”. Some acknowledged that being a role model was challenging because “I really like soda, so it can be hard to limit them” and “if they (children) see you drinking it (SSB), they want it”. Some parents also did not realize that some beverages were sweetened, “I used to give Gatorade, but the dentist said no”.

Busy schedules were another barrier that parents faced in curtailing SSB intake in that time scarcity enabled decisions that limited access to healthier alternatives. For example, parents reported feeling like they were “living at Wawa and Wendy’s during the sports season and school year” where sugary drink options were common and healthy alternatives were limited. Fast food restaurants, gas stations, and convenience “stores have such a small selection of water and then huge selections of sugary drinks; so every time we are in the gas station or something, he asks for a pop”. Parents also raised the concern of limited availability of healthy alternatives at after school events, remarking “school-related activities don’t always have a lot of water and you can only find pop and sweetened tea at sporting events”.

Parents felt that SSB intake had increased as children got older. One parent stated, “his consumption has increased exponentially” and another reported “now they drink them (SSB) more; when they were younger, they didn’t drink them. I even find bottles of soda hidden under their beds now”. Children’s growing maturity and independence was seen as an obstacle to limiting SSB intake. As kids got older, they were purchasing lunch more often and parents were “unsure of how much sugary beverages they drink when they buy lunches at school”. And, ”it is more difficult to control the consumption of juices and sugar (when kids are at school)”. Parents felt kids’ beverage choices at home were influenced by school experiences, noting that “the school gives the option for flavored milk”, so now they have “had to switch to chocolate or strawberry milk (at home)”. Another parent reported that when “other parents give sugary drinks for school lunches… my kid says, ‘why can’t I bring that, too?’”.

Another aspect of children’s increasing age that got in the way of keeping SSB intake in check was that, as they got older, they “have more involvements, which often mean more celebrations and sugar intake”. They attend more “school activities, friends’ birthday parties, and get togethers” where soda was readily available. One parent commented, “I blame little league baseball—he used to get a hotdog and a soda after a game; he had never had it (SSB) until he was 8 years old”.

SSB purchases of other family members also presented roadblocks to limiting intake of these beverages. “In my house the only problem is that my sister lives with us and she will buy soda and then they (kids) will drink it. I can’t stop her from buying soda because she is an adult, since they see her drinking it they will ask her for soda and drink it”. Responsibilities outside the home presented another obstacle because parents were not able to supervise children’s consumption of sugary beverages during these times: “It’s difficult to limit their drink consumption when I’m not home because they will get what they want”. Another obstacle, television, made it “more complicated” for parents to control children’s SSB intake because it provided frequent exposure to advertisements for these drinks.

#### 3.1.3. Parents’ Strategies for Overcoming Barriers to Limiting SSB Consumption

The number one strategy for limiting sugary beverage intake was environmental control: “keeping them (SSB) out of the house” and “don’t buy them”. Another common approach to regulating SSB consumption was to “set boundaries” and reserve sugary drinks for “special occasions”. For instance, “Friday night is pizza night and my children get a soda beverage, but it’s not (a) regular (occurrence)”. Occasional intake of SSBs was recognized by some parents as being important for teaching self-control, because “you cannot forbid her to do it or else she will go crazy later when it’s available”. “My basic rule is water or milk, but exceptions at parties or events with other kids. They probably have it like 3 times a month—so not often, but they have learned what they should have”. However, other parents were more rigid in their approach (e.g., “I force them to drink water; if they don’t want water, then they don’t get anything” and “I distribute (SSB0, and I use child-locks for all of my pantries”).

Making “milk and water the drinks of choice” in their homes and being sure “kids grew up with the mind set to enjoy and prefer water” were other methods parents used to limit SSB intake. To boost children’s preference for water, parents tried “cutting up lemons and limes in the water to give it a little punch”, serving “agua fresca” (water infused with fruit), and making “it more interesting … have fruit and ice in it” or add “sugar-free flavored water packets”. To encourage healthier drink options, parents also proposed environmental solutions. For example, “having an accessible fridge where they can reach for water”, “keeping a large Brita filter in the refrigerator” and having “an array of water bottles” and “fun cups for the kids to drink out of” were identified as useful tools for encouraging water intake over SSB.

Another approach was to lower sugar intake from beverages. Parents “cut juice with water”. They also reduced the sugar content of flavored milk by “putting in mostly white milk with just a little chocolate (drink mix)”. Parents also touted the benefits of diet soft drinks to children, describing them as “sugar-free flavored water” and seltzer water as healthy alternatives to sugary drinks. One parent used portion control as a means to control SSB intake: “get them a little glass if they ask”.

Promoting various qualities of beverages was another tactic parents employed to limit SSB intake. For instance, the health benefits of alternative beverage options were conveyed when parents reminded children that “milk helps bones”, “milk will make their teeth white”, and will help you “get stronger and taller”. Parents also promoted the benefits of water. “When you do drink water, when your kids are well hydrated, it shows in everything—your eyes aren’t as puffy, your skin feels clearer—(this) translates over to kids. When your kids are hydrated, they aren’t as sluggish and it keeps your body at its best. Sharing that with the kids keeps them at their best”. Some parents helped children think about calories from SSB by telling them if they “do not have soda, they can use those calories to have something more enjoyable”. Thirst quenching abilities also were invoked (“juice and soda are going to make you thirstier”). Others pointed out that “it’s helpful to have the orthodontist and dentist” endorse milk and water and the importance of limiting SSBs.

Many parents recognized that “setting a good example” was an effective way to help kids keep SSB intake under control. One mother observed, “seeing me drink water helps them. The mirrored behavior is important”. Parents also were motivated to drink more water themselves and encourage their children to do the same because, “your body feels better when you drink water and you feel better about yourself”.

### 3.2. Children’s Focus Groups

Survey results indicated that children had an average intake of 2.13 ± 2.52 SD servings weekly of SSBs. A review of children’s responses indicated that SSB intake was similar across age and state of residence. Focus group data were similar across age groups and state, so data were combined.

#### 3.2.1. Children’s Attitudes toward SSB Consumption

Kids reported drinking a wide array of SSBs, with intake ranging from infrequent (“we don’t have them often, maybe once a month”) to often (“I have them once a day or I have a little more than that”). These beverages were consumed at meals or alone. Several kids reported having SSBs more frequently on the weekend and at events like birthday parties and when going out to eat. Some participants indicated “you can only drink it sometimes, but not a lot”.

Reasons many children gave for limiting consumption of SSBs were “it’s very important because if you drink too much, it is not good for you” and “sugar is kind of bad for you” “because might have too much energy, get sugar rushes” and makes them “hyper” and “hard to go to sleep”. Health effects were common explanations children gave for limiting SSBs; these ranged from general (they could “get a stomachache” or “get sick”) to more specific health effects (sugary drinks are “bad for your teeth”, “not good for your skin”, “not good for your kidneys”, or “are not good for your health because they can cause diabetes”). Some commented that SSBs are “bad for your bodies because of the calories, I think” and “you can also start getting fat”.

A few children linked calories in SSB with energy expenditure and discounted the importance of limiting SSBs if balanced with exercise, “I ran yesterday, so I don’t need to limit it (SSB)”. One commented, “it is less important for us (to limit SSBs) than for them (parents) because they don’t burn a lot of calories like we do”.

Some children commented on hydrating qualities of drinks. “When I play outside, I drink water instead of sugary drinks because you cramp if you don’t have water and have too much sugary drinks” and understood that “it is important to drink a lot of water or you will get dehydrated”. Kids also connected SSB consumption to decreased athletic performance and energy levels saying, “when I go to recess, I will run slower”.

Although most children felt keeping SSB intake under control was important, some did not. “I don’t think it’s important because I love sugar, and it tastes good”.

#### 3.2.2. Children’s Perception of Their Parents’ Attitudes toward SSB Consumption

Most children thought parents felt it was important to limit SSB intake because parents tell them, “if you drink a lot of it (SSB), you can get sick” and that parents “get happy if you don’t drink a lot of sugary drinks” but “get mad if I drink too much”. Other ways parents conveyed to children that it was important to control sugary beverages was that parents told kids “not to drink a lot of them”, set limits on the number of SSBs kids can drink (“My mom says to only have one can of soda a day”), controlled access (“my parents only buy 1 or 2 (boxes of pop), but they put it somewhere where I can’t reach or see it”), set “rules requiring us to drink water or milk”, and rewarded kids for drinking water.

Others concluded that parents believed it was important to limit SSB because parents promoted healthier beverages “she (mom) fills up big pitchers with water and we have to drink that”, or “mom and dad set goals not to buy them”. Conversely, parental behaviors led one child to surmise, “dad doesn’t care, he drinks soda”.

#### 3.2.3. Children’s Perceived Barriers to Limiting SSB Consumption

Although most kids understood the importance of limiting SSB intake, they noted many barriers hindered their efforts. A commonly mentioned theme was parental influence. Many children indicated that they want to do what their parents do—“I want what my parents have. When my parents drink pop, I want it, too”. Another child remarked, “I have iced tea that my dad drinks a lot and sometimes I steal it and drink it”. Another participant indicated, “every time my mom drinks soda, we see it and then we get jealous”. Parental behaviors were not limited to sugary drinks: “it (soda) does make me want to have what they have. But if they have milk or water, I’ll drink it, too”.

Another common barrier to limiting sugary drinks was the taste. “I drink soda because it tastes good. I’m not judging water because it has no taste, it’s just clear”. There also was a social aspect that made limiting SSBs difficult as well. Many children reported that “I usually have them (SSB) at parties” or on “special occasions, like birthdays and holidays”. Having their own money to buy sugary drinks presented a roadblock to curbing SSB intake for some. The home environment presented another obstacle: “we keep around 100 cans (of soda) at home in our basement”. Another barrier was wanting to be like siblings or friends, “my friend is allowed to drink whatever she wants” and using these beverages as a reward (we get) a little everyday if we have good behavior”).

#### 3.2.4. Children’s Strategies for Overcoming Barriers to SSB Consumption

Children indicated that parents play the largest role in helping them limit SSBs and gave various strategies parents could use. One common suggestion was for parents to not drink sugary beverages themselves—“tell parents not to drink a lot of soda” because “it makes me want it when they drink sugary drinks”. Role modeling healthy behavior was another strategy: “my mom drinks a lot of water so it makes me want to drink water.”

Teaching kids about sugary drinks was another approach children recommended parents try: “show your kids a video to educate them and tell them what these drinks are made of and why they are not healthy” or “have them (kids) write paragraphs of why they are drinking so much sugar”. Explaining the disadvantages of SSBs was another idea children had: “you still feel thirsty after drinking a sugary drink, but not if you drink water”, “if you drink it [after sports practice], then its wasting what you just worked off”, and “tell kids they could get sick” and “it’s not good for the kidneys”. On the other hand, some thought parents could explain the advantages of healthier drinks, and they could “explain it’s good for sports” and that “water is healthy”.

Children also proposed that parents impose limits on SSBs, such as “2 sugary drinks a day” or “only let children drink juice if they drink a lot of water” and “give us certain drink choices and we can only choose from those”. Another method kids thought parents should try was to promote milk and water, offer kids incentives (e.g., “if you drink water or milk, then you get a reward”), or when kids refuse water or milk, punish them (e.g., “put children in timeout because they will learn their lesson”).

To lower intake of sugary beverages, kids proposed making “it more difficult to get pop”, drinking more water (“water helps me drink less sugary drinks”), buying “soda without sugar in it” and “healthy drinks”, diluting juice with water, and making healthier options more appealing (“flavor the drinks or make something creative like smoothies”, “drink out of straws with loops”, “put raspberries in the drink”). Other strategies children thought parents could use were “have them [children] drink 3 bottles of water before they can drink sugary drinks” and “trick them–dye water to make it look like juice”.

Children also suggested environmental controls that restrict access to SSBs: “I’ve been to friends’ houses and they will have 5 to 8 boxes (of soda) at a time, but we don’t have a lot of boxes at a time–it’s easier when you don’t have as much around”. Other environmental controls were to hide sugary drinks, “just don’t buy it”, have healthy options available (“have lots of milk, instead”), and “get the good drinks for them and bring it to them”.

They also thought that small serving sizes (“don’t give them a lot”), limiting access to certain times or events (e.g., after practice, weekends), and setting goals for healthier beverages (“I aim for 8 bottles of water every day”) would be helpful ways to control SSB intake. Finally, kids recommended being role models themselves (“don’t drink sugary drinks around siblings…they are likely to see what you are drinking and would like that drink”).

## 4. Discussion

This study aimed to explore the cognitions, barriers, and facilitators related to SSB intake of school-age children and parents of school-age children. A second aim was to use the finding to create recommendations for future nutrition education programs targeting limited SSB consumption based on constructs from the Social Cognitive Theory (Table 1). Social Cognitive Theory [28,29,30] provides a useful framework for categorizing factors known to promote behavior change and thus was used to organize future programming recommendations.

The findings from this study reveal that most parents and children accurately perceive the negative health effects of excessive SSB intake. For instance, both parents and children knew that sugary beverages lacked important nutrients [40]. In addition, they accurately reported that SSBs can promote weight gain and contribute to health problems [41]. However, not all participants were fully cognizant of these negative health benefits, thereby highlighting an opportunity to expand their knowledge as well as reinforce the knowledge of those who are more informed.

Some parents and children were aware of the effect of SSB on oral health and mentioned dental health professionals as sources of SSB-related information. According to the American Academy of Pediatric Dentistry, frequent consumption of SSBs increases the risk of dental caries [42]. Helping parents and children fully realize the potential outcomes of SSB on oral health could be an important component of future nutrition education programming to elicit reductions in sugary beverage intake. Furthermore, the American Dental Association recognizes the role of dental professionals in promoting healthy lifestyles and behavior change to reduce the incidence of obesity by collaborating with other health care professionals and organizations [43]. General oral health recommendations include visiting the dentist twice a year, which provides an opportunity for nutrition education professionals to develop theory-based training programs to train oral health professionals to discuss limiting SSBs with families and provide educational materials and training that oral health professionals can provide to families to help them limit SSB consumption in children [43].

A negative outcome expectation of SSB consumption shared by some parents and children was hyperactivity. Although this belief is commonly held by many consumers, the effect of sugar on behavior in children remains unclear. Although a landmark 1995 meta-analysis published in the Journal of the American Medical Association concluded that sugar did not have an effect on behavior [44], more recent cross-sectional data suggest that risk for hyperactivity/inattention does rise as SSB consumption increases [44]. Helping parents and kids understand that SSB increase may affect behavior in some people could help them implement behaviors to curb intake of these drinks.

Participants’ observation that SSB consumption affects sleep patterns is supported by research reporting links between dietary sugar and sleep duration [45]. The 2014 Sleep in America^®^ Poll revealed that nearly one in three children age 6 to 11 years in the Unites States gets 8 h or less of sleep each night [46], falling short of the National Sleep Foundation’s recommended 9 to 11 h per night [47]. The link between short sleep duration and increased BMI is well established [48]. Decreased sleep duration associated with SSB consumption may further exacerbate the association between SSB consumption and BMI.

Parents were also correct to express concern about the effect of caffeine in SSBs on children’s behavior. Three-quarters of the young children in a study evaluating caffeine consumption were caffeine consumers, and caffeine consumption was negatively correlated with average number of sleep hours [49]. In addition, caffeine can have negative cardiovascular effects in children [50]. While caffeinated soda consumption in children has declined over the years, more children are consuming trendy coffee and energy drinks high in sugar and caffeine [51]. Future nutrition education programs should build knowledge of the negative effects of caffeine from SSB and offer strategies for limiting caffeine consumption.

Although SSB consumption reported by participants did not differ by geographic location, parents in West Virginia commented that SSB consumption was a normal part of their lifestyle and culture, which they felt made it especially difficult to decrease consumption. The Theory of Reasoned Action postulates that an individual’s behaviors are influenced by perceived social norms, or the extent to which they believe others support engaging in a particular behavior [52]. Similarly, the construct of reciprocal determinism from SCT suggests that individuals’ behaviors are influenced by their environment, including cultural influences [29]. The potential effect of geography and culture on SSB intake is important to consider in nutrition education programs. Cultural norms can both precipitate and reinforce positive, as well as negative behaviors. For example, a study aiming to improve adolescent normative beliefs related to smoking found that students’ perceived prevalence of smoking was linked to their risk of smoking, as was their beliefs that smoking was popular among “successful/elite elements of society” [53]. The study also found that disapproval of smoking by friends and family was significantly negatively associated with adolescent smoking behaviors [53]. Future interventions may promote behavior change by aiming to alter normative beliefs related to SSB consumption.

A common barrier to limiting children’s SSB intake was parental role modeling. Children tend to model their parents’ eating behaviors, lifestyle, eating-related attitudes, and body image satisfaction [54]. In the current study, both parents and kids recognized that children were affected by parent actions. In fact, children suggested parents not drink as much SSB as a strategy to help kids decrease their own intake, however some parents indicated that it was a struggle to not consume SSBs. Several studies have demonstrated a positive association between parent knowledge about diet and health and positive parental influence on children’s beverage intake [31,55,56,57], thereby highlighting the importance of building parent knowledge of the importance of limiting SSBs to motivate them to limit child SSB intake. Often parents reported turning to quick, sugary beverage options due to the time constraints and limited healthy options at convenience stores. Providing strategies for healthy beverage options on-the-go as part of nutrition education interventions could enable families to reduce SSB intake, as would building parents’ time management skills.

Another barrier noted was parents’ lack of knowledge of the school food environment and policies that promote healthy eating in this setting. It was evident from the focus groups that many parents were not aware of federal guidelines designed to ensure school beverage options are nutrient dense [58]. Instead, parents felt that their children’s beverage choices at home were negatively influenced by school experiences. Studies consistently show that students who participate in the National School Lunch Program (NSLP) consume more fruits, vegetables, and milk and less SSBs and low-nutrient, energy-dense items than non-participants [59,60]. A cross-sectional study exploring the relationship between school lunch participation and dietary patterns also indicated that children who participated in NSLP consumed less than one-third the average amount of energy from SSBs as non-participants [59].

Findings from the current study suggest parental disapproval of flavored milk in the school setting. Federal guidelines allow non-fat flavored milk as part of the NSLP, which has been endorsed by the American Academy of Pediatrics [61] and the Academy of Nutrition and Dietetics [62,63] as part of an overall healthy diet. Additionally, a recent systematic review reported that flavored milk increases overall milk intake by 28% [64]. Most other SSBs contain more added sugar than flavored milks, which provides an opportunity to teach parents and children to make better SSB choices, such as flavored milk in moderation when away from home.

Both children and parents recognized the importance of the home food environment in promoting healthy beverage choices and suggested not purchasing SSBs as a key strategy to limiting consumption [25,62,65]. Similarly, in a qualitative study with Latino adolescents and their parents, youth cited home availability as a key factor driving their SSB consumption [66]. Research supports the effectiveness of this strategy in that the availability of soft drinks in the home is strongly associated with soft drink consumption. Future nutrition interventions should promote SSBs as a beverage to consume on occasion rather than every day and should suggest that SSBs be purchased at the time of consumption rather than kept on-hand in the house.

Both parents and children in the study reported here indicated the importance of setting boundaries for SSB intake. Recommendations by children that parents incentivize or punish children as a means for promoting healthier beverage intake suggests kids have experienced non-recommended authoritarian parental feeding practices. In general, an authoritative feeding style, where parents use supportive and responsive feeding practices to encourage heathy eating, is associated with healthier dietary behaviors and BMI [67,68], although a more restrictive parental feeding style was associated with less soft drink consumption in teenagers [69]. Helping parents and children recognize the benefits of authoritative feeding styles could help families now, as well as future families of the current generation of children, form healthier dietary habits, including limited SSB intake.

Previous research has shown that interventions that focus on environmental changes are most successful at reducing SSB consumption in school-age children [70]. This is particularly true for interventions that target the home rather than school environment [71]. Interventions aimed at teaching parents of school-aged children to set goals for making health-related behavior changes for their families can be effective at reducing children’s SSB consumption [72]. The success of these interventions is congruent with some recommendations in Table 1, including facilitating behavior change by adjusting the home environment and building parent self-efficacy for making behavior changes.

## 5. Conclusions

This qualitative research provides important information regarding parents’ and children’s cognitions (e.g., beliefs, attitudes), barriers, and facilitators related to children’s SSB consumption. To the authors’ knowledge, this study is the first to qualitatively explore SSB cognitions and use them to generate recommendations for future nutrition programming aiming to lower SSB intake. However, the small sample size limits the ability to detect ethnic differences in study participants. Furthermore, self-selection bias may have resulted in a sample that is not representative of the general population.

Achieving the goal of limited SSB consumption is important given that SSBs are a significant source of added sugars in the diets of children and are considered an important contributor to the obesity epidemic [73]. Educating both parents and children is important because parents serve as household food gatekeepers and children’s role models and, as children age, they have more opportunities to make their own beverage choices. Future research should aim to implement the recommendations generated by this study in interventions and assess their effectiveness in helping families reach the goal of reduced SSB intake and associated health outcomes.

## Figures and Tables

**Table 1:** Recommendations for Interventions Targeting Limiting Sugar-Sweetened Beverage Intake in Families with School-Age Children.

Social Cognitive Theory Recommendations for Future Interventions Promoting Reduced Sugar Sweetened Beverage (SSB) Intake	
Outcome Expectations	Expand SSB outcome expectations to include weight management and oral health.
Outcome Expectations	Expand SSB outcome expectations to include the negative effect of caffeine in coffee and energy drinks.
Reciprocal Determinism	Teach parents and children about actual SSB behaviors to improve their perceived norms of this behavior.
Outcome Expectations	Expand SSB outcome expectations to include the positive effects of choosing flavored milk in moderation over other SSBs.
Facilitation	Provide oral health professionals with nutrition education materials and training to enable them to help families decrease SSB intake.
Facilitation	Provide parents with strategies to break from geographic and/or cultural norms that encourage SSB consumption.
Facilitation	Provide opportunities for parents to develop strategies that control SSB availability in the home environment, such as limiting amount of SSB on hand and purchasing them only at the time of consumption.
Facilitation	Provide parents with opportunities to develop more authoritative parental feeding skills and an understanding of how this type of parenting can facilitate child development.
Facilitation	Inform parents about school meal program guidelines and policies and encourage them to visit children’s school cafeteria.
Facilitation	Provide tips for identifying healthier, on-the-go beverage options, including planning ahead for busy days.
Facilitation	Share quick and easy ways to incorporate healthy beverages in meals and snacks.
Facilitation	Provide parents with time management strategies.
Observational Learning	Explain the importance of healthy role modeling to parents and encourage parents to make better beverage choices to demonstrate healthier beverage choices to children.
Self-efficacy	Build parents’ confidence in their ability to provide healthier alternatives to SSBs and enjoy more nutrient-dense beverage.
